# Selenoprotein S ablation-mediated pyroptosis contributes to liver damage resulting from selenium deficiency in chickens

**DOI:** 10.1016/j.psj.2025.105269

**Published:** 2025-05-05

**Authors:** Huanqi Zhang, Xiaozhe Chen, Tingjin Lu, Qiyuan Cao, Xiaojing Li

**Affiliations:** College of Animal Science and Technology, Northeast Agricultural University, Harbin 150030, PR China

**Keywords:** Selenium, Selenoprotein S, Pyroptosis

## Abstract

Selenium is an essential trace element for the synthesis of selenocysteine. Selenoprotein S (SELS) acts as a carrier protein for selenium and exhibits anti-inflammatory properties. However, the role of the SELS in selenium deficiency remains unclear. This study aimed to investigate the role of SELS in selenium deficiency-mediated pyroptosis. A selenium-deficient chicken model was established using a low-selenium diet, allowing for analysis of the pyroptosis markers GSDMD and NLRP3 by immunohistochemistry and the expression levels of 25 selenoproteins in the liver. The results show that the selenium-deficient diet increased the levels of NLRP3 and GSDMD while reducing the expression of nine selenoproteins (DIO1, GPX1, GPX6, TXRD2, SELF, SELN, SELO, SELS, and SELT). SELS ablation abolished the activities of antioxidant enzymes, leading to excessive production of ROS and MDA. In addition, SELS knockdown activated the NF-κB pathway and induced pyroptosis. Following transfection, the introduction of N-acetylcysteine, BAY11-7082, or MCC950 alleviated the pyroptosis induced by SELS knockdown. However, MCC950 did not affect the NF-κB pathway, and both BAY 11-7082 and MCC950 were ineffective in reducing ROS accumulation. In conclusion, SELS deficiency leads to ROS generation and activation of the NF-κB pathway activation, ultimately inducing pyroptosis and the release of inflammatory factors.

## Introduction

Selenium is an essential microelement that plays important roles in enhancing immunity, improving antioxidant capacity, and alleviating the toxicity of harmful substances in organisms (Zhang et al., 2022a). The liver is the organ that is most sensitive to selenium supply and is the central organ that regulates the distribution of selenium throughout the body (Speckmann et al., 2017). Selenium deficiency induces oxidative stress, inflammation and pyroptosis in hepatocytes (Burk et al., 2008; Gu et al., 2022). Rats fed a selenium-deficient diet developed hepatic necrosis and increased lactate dehydrogenase as well as alanine aminotransferase serum levels, accompanied by the production of abundant oxidants (Fraga et al., 1987). In livestock and poultry breeding, dietary selenium supplementation alleviates liver metabolic disorders induced by chronic heat stress (Liu et al., 2021). Moreover, Tang et al. (2020) found that leakage of selenium induces redox imbalance, metabolic reprogramming, and inflammation in the liver (Tang et al., 2020). In cow breeding, selenium deficiency decreases the activities of antioxidant enzymes and increases the accumulation of oxidation products in the calf liver, which induces the expression of inflammation-related factors, ultimately leading to liver injury (Wang et al., 2022). In poultry, liver necrosis occurs when chicks are fed a selenium-deficient diet (Li et al., 2021). In addition, oxidative stress and endoplasmic reticulum stress are involved in the apoptosis and autophagy of chicken hepatocytes in selenium-deficient liver injury (Wang et al., 2017; Yao et al., 2015). Taken together, there is ample indication that selenium affects liver function in several ways.

Selenium exerts its biological functions primarily via selenoproteins (Bai et al., 2025). Numerous characterized selenoproteins are associated with redox reactions and play a role in resisting oxidative damage via various pathways (Labunskyy et al., 2014). In chicken liver cells, yeast-derived selenium enhances the antioxidant effects by elevating selenoproteins levels and counteracting cadmium-induced pyroptosis (Xing et al., 2022). Accordingly, selenium supplementation activates the expression and synthesis of selenoproteins, which can alleviate the oxidative damage induced by diseases, stress, and genetic factors (Guo et al., 2021). Selenium deficiency disrupts redox signaling of selenoproteins by affecting their transcription levels (Legrain et al., 2014). The selenoproteins glutathione peroxidase and thioredoxin reductase primarily scavenge lipid peroxides (Zhang et al., 2020). Endoplasmic reticulum membrane SELS and SELK alleviate oxidative stress by restoring endoplasmic reticulum stress caused by misfolded proteins (Jing et al., 2023). In addition, studies involving different species have revealed that inhibition of SELS leads to increased levels of the inflammatory cytokines IL-6 and TNF-α, suggesting that SELS is crucial for regulating cytokine production in macrophages and controlling the inflammatory response (Curran et al., 2005). Moreover, SELS knockdown effectively negates the protective effect of selenium supplementation against inflammatory liver injury in mice (Liu et al., 2022).

Cell death is as a key indicator of tissue injury, indicating the extent of liver damage at the cellular level, and typically relies on primary hepatocytes, which provide a reliable reflection of hepatic in vivo conditions (Vilas-Boas et al., 2019). Pyroptosis is an inflammation-induced programmed cell death. However, excessive pyroptotic cells can lead to structural and functional damage to organs, a process closely linked to the occurrence and progression of liver diseases, such as non-alcoholic liver disease, liver fibrosis, and drug-induced liver injury (Franchi et al., 2009; Xia et al., 2019; Zhang et al., 2023). In the canonical pyroptotic pathway, caspase-1 is activated by the NLRP3 inflammasome (Aachoui et al., 2013), followed rapidly by the activated caspase-1 cleaving the substrate gasdermin D (GSDMD), leading to the cleavage of pro-IL-1β and pro-IL-18, which induces pyroptosis and the release of inflammatory factors, thereby causing tissue inflammation (Dou et al., 2022). NF-κB and ROS usually play important roles in inflammatory responses and pyroptosis (Gong et al., 2018; Yu et al., 2017). For instance, NF-κB promotes the transcription of a variety of pro-inflammatory cell mediators, including NLRP3, pro-IL-1β, and pro-IL-18 (Koushki et al., 2021). Several substances can activate the NF-κB/NLRP3 pathway, such as carbon tetrachloride, which leading to pyroptosis and tissue inflammation in murine liver cells (Kim et al., 2023). Furthermore, inhibition of NLRP3 reduces ROS levels, and inhibition of ROS levels reduces NLRP3 production, accompanied by a reduction in pyroptosis (Wang et al., 2021a). This suggests a potential interaction between NLRP3 and ROS that may contribute to pyroptosis; however, the specific mechanism remains unclear.

In summary, although high levels of SELS have been identified in the liver, the precise function of SELS remains poorly understood, particularly the mechanisms by which it regulates hepatocyte pyroptosis. Therefore, we developed an in vivo model involving chickens fed a selenium-deficient diet, a SELS knockdown model using primary hepatocytes, and a chicken leghorn male hepatoma cell line (LMH). The expression of 25 selenoproteins, the occurrence of pyroptosis, pyroptosis marker genes and related signal transduction pathways, oxidative stress, and the NF-κB pathway were assayed. The results of this investigation may clarify the role and mechanism of SELS in regulating pyroptosis and selenium deficiency-induced liver injury, thereby providing a reference for expanding the biological functions of selenium and preventing and controlling selenium deficiency diseases.

## Materials and methods

### Animals

Animal experiments were performed with the approval of the Institutional Animal Care and Use Committee of Northeast Agricultural University (SRM-11). Broilers were purchased from Harbin Xinghua Breeder Farm Huiyang Bearded breed. Thirty 1-day-old chickens were randomly divided into a control group and a low-selenium (low-se) group, with 15 chickens in each group randomly divided into three cages housing 5 chickens each. The control group was fed a normal diet (Se content: 0.278 mg/kg) and the low-se group was fed a selenium-deficient diet (Se content: 0.039 mg/kg) (Shi et al., 2023). Throughout the experimental period, the chickens were allowed ad libitum access to food and water. After 40 days of breeding, the chickens were euthanized. The liver tissues were isolated and stored in *a* − 80 °C refrigerator or 4 % paraformaldehyde at 4 °C.

### Immunohistochemistry

The liver tissues stored in 4 % polyformaldehyde were subjected to gradient alcohol dehydration and xylene to enhance the transparency and then embedded in paraffin. The paraffin sections were placed in an environmentally friendly dewaxing solution (Servicebio, G1128) and anhydrous ethanol for dewaxing. Tris-EDTA antigen repair solution (Servicebio, G1206) was used for antigen thermal repair for 30 min, followed by the addition of 3 % hydrogen peroxide solution and incubation at 27 °C for 25 min in the dark to block endogenous peroxidases. After blocking with 5 % BSA for 30 min, primary antibodies against NLRP3 (ABclonal, A12694, 1:100) or GSDMD (ABclonal, A24476, 1:200) were added overnight at 4 °C followed by incubation with HRP-conjugated secondary antibody (Servicebio, GB23303, 1:200) for 45 min. After treatment with 3,3′-diaminobenzidine solution (Servicebio, G1212), images were captured using a fluorescence microscope. Three images were randomly selected from each group (Control and Low-se), and the ImageJ IHC Profiler plug-in was used to determine the percentage of NLRP3- and GSDMD-positive cells.

### Extraction of primary hepatocytes

Specific pathogen-free chicken embryos (age: 12 days) were provided by the Harbin Veterinary Research Institute (Harbin, China). The liver tissue was peeled and cut into 1–2 mm pieces. After three washes, 0.1 % IV collagenase (Biotopped, CG6160C) was added and the tissue was digested at 37°C for 30 min. The digestion was stopped with a solution containing 84 % DMEM (Gibco, USA) + 15 % FBS (Gibco, USA) and 1 % penicillin–streptomycin. The cells were centrifuged at 800 rpm (112 × *g*) for 5 min, resuspended at 37 °C, and cultured in 5 % carbon dioxide.

### Cell culture and transfection

LMH cells (ATCC, USA) were cultured in RPMI1640 (Gibco, USA) supplemented with 10 % FBS at 37 °C in 5 % CO_2_. Primary hepatocytes were cultured in DMEM (Gibco, USA) supplemented with 15 % FBS at 37 °C in 5 % CO_2_. The cells were then transfected with 3 μL of 20 μM si-SELS (Ribobio, China) and 3 μL of Lipofectamine™ RNAiMAX Reagent (Invitrogen, USA) in 1 mL of Opti-MEM™ (Gibco, USA). The primary hepatocytes and LMH cells were treated with 10 mM NAC (Sigma-Aldrich), 10 mM MCC950 (MCE), or 1 mM BAY11-7082 (MCE) for 24 h. The si-SELS sequence information: 5′-CAUGCAAGAAGGCAGAAGUUACAAA-3′.

### Detection of oxidative stress

Primary hepatocytes and LMH cells were incubated with medium containing 10 μM DCFH-DA probe (Nanjing Jiancheng, China, E004-1-1) for 30 min, and the intracellular ROS content was evaluated using a fluorescence microscope (Thermo Fisher Scientific, USA). Quantitative processing was performed using ImageJ software. The levels of T-AOC, T-SOD, GSH-px, CAT, and MDA in primary hepatocytes and LMH cells were detected according to kit instructions, and a histogram was generated using GraphPad Prism 10.4.0 software. The kits were obtained from the Nanjing Jiancheng Bioengineering Institute. Each sample was measured three times.

### Immunofluorescence

Cells were fixed with 4 % paraformaldehyde and treated with 5 % BSA solution for 1 h and then incubated overnight at 4°C with antibodies against NLRP3 (Abclonal, 1:500) and GSDMD (Abclonal, 1:200) followed by Alexa Fluor® 488 and 594 secondary antibodies goat anti-rabbit IgG (Biodragon; 1:500) for 1 h at room temperature in the dark. Nuclei were stained with DAPI (Beyotime) for 5 min. Three images of each sample were captured using a fluorescence microscope (Thermo Fisher Scientific) at 200 × magnification.

### Western blot

Western blotting was performed as described previously (Li et al., 2024). The membranes were blocked with Minute Block (AIWB-004, Affinibody LifeScience Co.Ltd, China) at 37°C for 10 min and washed twice. The membranes were then incubated overnight with primary antibodies. The primary antibodies used in this study were anti-SELS (Sigma-Aldrich, 1:1000); anti-NLRP3 (Abclonal, 1:1000); anti-caspase-1 (Abclonal, 1:1000); anti-GSDMD (Abclonal, 1:1000); anti-IL-18 (Abmart, 1:1000); anti-IL-1β (Abclonal, 1:1500); anti-NF-κB (Beyotime, 1:1000); anti-COX-2 (Wanleibio, 1:1000); and anti-β-actin (ABclonal, 1:3000), incubated overnight. After washing thrice for 10 min each, anti-mouse and anti-rabbit IgG (Immunoway, 1:10000) were used as secondary antibodies for 1 h. Image J software was used to quantify the protein expression levels in each group. The results are reflected by grayscale analysis using β-actin as an internal standard.

### Quantitative real-time polymerase chain reaction (qRT-PCR)

RNA was extracted using TRIzol® reagent (Invitrogen, USA) and then reverse transcribed into cDNA (Bioer, China). SYBR® Green dye (Bioer, China) was used for qRT-PCR in a LightCycler® 480 II system (Roche, Switzerland). The RNA concentration and purity (260/280 ratio) were detected with Microvolume UV-Vis Spectrophotometer (BioDrop, UK). The cDNA template (20 μL system) was synthesized based on 1 μg of total RNA. The reaction system consisted of 0.2 μL forward primer, 0.2 μL reverse primer, 5 μL SYBR® Green, 1 μL cDNA template and 3.6 μL nuclease-free water. The reaction program was one cycle of the first step: 95 °C for 2 min, and 40 cycles of the second step: 95 °C for 5 s followed by 60 °C for 15 s. The primer sequences are listed in Table S1. The mRNA expression relative to β-actin was calculated using the 2^-ΔΔCT^ method (Livak and Schmittgen, 2001). The qRT-PCR was performed in triplicate for each sample.

### Network analysis

The PPI network diagram of the selenotranscriptome and pyroptosis-related genes was constructed using the STRING database (https://cn.string-db.org/). The relative expression of mRNA in the selenotranscriptome was analyzed by principal component analysis (PCA) using Origin 2021 software.

### Statistical analysis

All data in this study were visualized in GraphPad Prism 10.4.0 in the form of mean ± standard deviation and normality tests (alpha=0.05). The fluorescence map was visualized using ImageJ software. The *t*-test was used to analyze the differences between the two groups. A *p* < 0.05 was considered statistically significant compared to the control group. A *p* > 0.05 was considered not statistically different and is indicated as ns. One-way ANOVA was conducted to compare the differences between multiple groups. Bars labeled with different letters indicate significant differences (*p* < 0.05).

## Results

### Selenium-deficient diet induces SELS ablation and pyroptosis

**Fig. 1:**
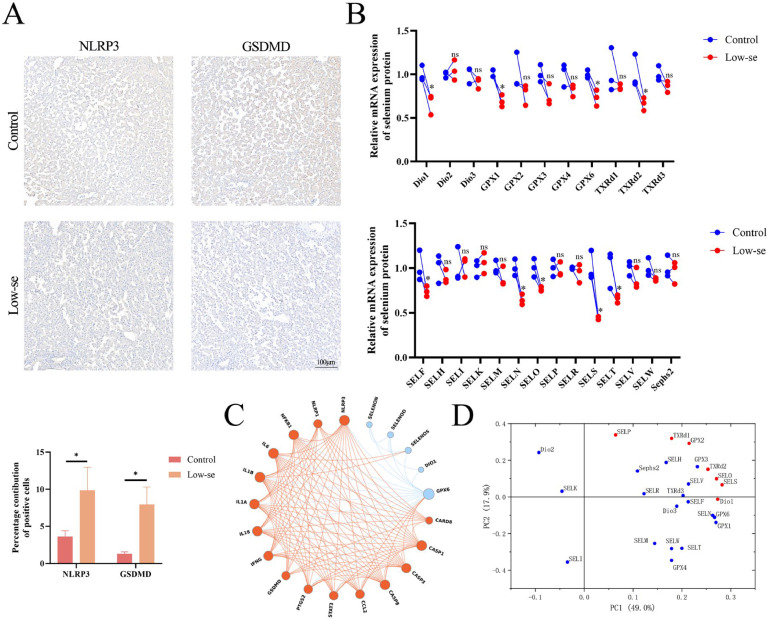
Selenium deficiency diet causes liver injury in chickens. (A) Immunohistochemical images and quantification of chicken liver (*n* = 3). (B) QRT-PCR was used to detect the transcriptional changes of 25 selenoproteins in chicken liver (*n* = 3). (C) PPI network diagram. (D) Principal component analysis of the selenotranscriptome. All data were biologically replicated (*n* = 3). A significant difference "*p* < 0.05″ is indicated by "*", otherwise "*p* > 0.05″ is labeled "ns", when compared with control group using t-test.

Our research group previously observed damaged liver tissue by H&E staining in chickens fed selenium-deficient diets (Shi et al., 2023). To further clarify the mechanism of liver injury induced by selenium deficiency, immunohistochemical analysis was performed, which showed that a low-selenium diet substantially increased the number of NLRP3- and GSDMD-positive cells in chicken liver tissue (Fig. 1A). This indicates that pyroptosis and selenium-deficient liver injury occurred concomitantly. We then evaluated the mRNA expression of 25 selenoproteins in chicken liver tissues after feeding a selenium-deficient diet. Compared with the control group, the mRNA expression levels of nine selenoproteins DIO1, GPX1, GPX6, TXRD2, SELF, SELN, SELO, SELS, and SELT were significantly decreased (p < 0.05), with SELS showing the lowest expression in chicken liver (Fig. 1B). The results of the protein–protein interaction (PPI) network analysis indicated that only GPX6 and SELS exhibited potential interactions with inflammation-related genes among these nine selenoproteins (Fig. 1C). PCA distinguished the major selenogens affected by selenium-deficient diets, and the results show seven (7) selenogens (DIO1, GPX2, TXRD1, TXRD2, SELO, SELP, and SELS) at a two-dimensional distance (Fig. 1D). This implies that SELS may play an important role in chicken liver injury upon selenium deficiency.

### SELS knockdown leads to hepatocyte pyroptosis

**Fig. 2:**
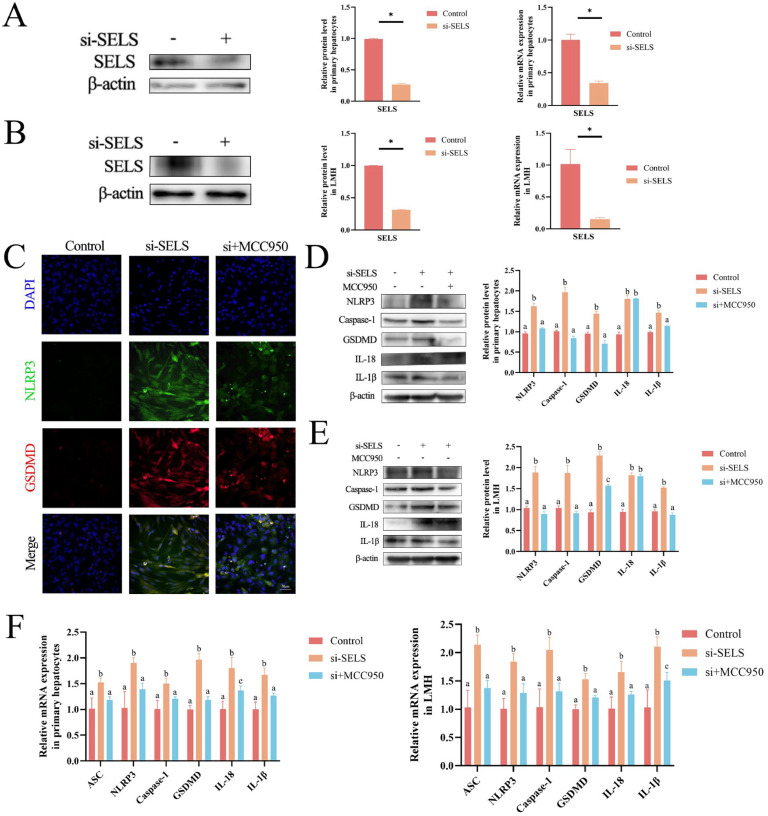
SELS knockdown induces pyroptosis. (A-B) Western blot and qRT-PCR shows protein and transcription levels of SELS in primary hepatocytes and LMH cells. (C) Immunofluorescence images and quantification of NLRP3 and GSDMD in primary hepatocytes. (D-E) Western blot shows protein levels of pyroptosis-related genes in primary hepatocytes and LMH cells. (F) QRT-PCR detects transcript levels of pyroptosis-related genes in primary hepatocytes and LMH cells. All data were biologically replicated (*n* = 3). A significant difference "*p* < 0.05″ is indicated by "*", otherwise "*p* > 0.05″ is labeled "ns", when compared with control group using t-test. Bars labeled with different letters indicate significant differences screened by one-way ANOVA.

To further confirm the effect of SELS on selenium-deficient pyroptosis in the chicken liver, we transfected specific siRNAs into chicken primary hepatocytes and LMH cells, which resulted in a significant reduction in SELS at the mRNA and protein levels (Figs. 2A–B). Protein expression in these two cell types was reduced by 69.5 % and 73.5 %, respectively, indicating that the knockdown model was successfully established. NLRP3 is involved in the initiation of pyroptosis, whereas GSDMD is involved in the execution of pyroptosis (Guan et al., 2024). Immunofluorescence analysis showed that the fluorescence intensities of NLRP3 and GSDMD increased significantly (p < 0.05) after SELS knockdown. The NLRP3 inhibitor MCC950 reduced the fluorescence intensity of NLRP3 and GSDMD after SELS knockdown in primary hepatocytes (Fig. 2C) and LMH cells (Fig. S1). In addition, the expression levels of pyroptosis-related genes, including *ASC, NLRP3*, caspase-1, *GSDMD, IL-18*, and *IL-1β*, were significantly higher (p < 0.05) than those in the control group after SELS knockdown in primary hepatocytes and LMH cells (Figs. 2D–F). The addition of MCC950 after SELS knockdown reduced (p < 0.05) the expression of most of the pyroptosis-related genes.

### The NF-κB pathway is downstream of SELS silencing

**Fig. 3:**
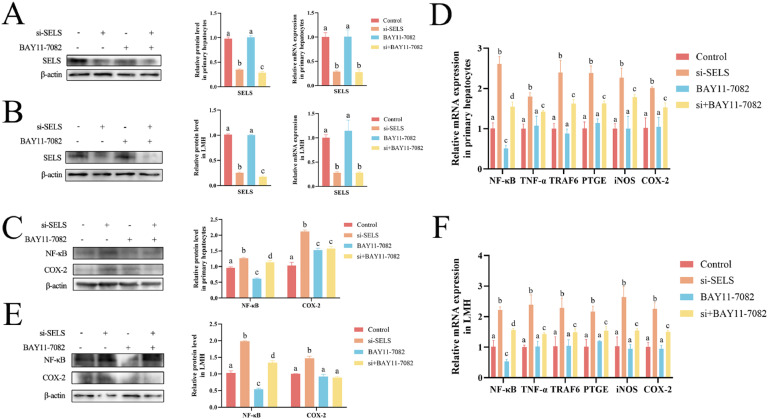
SELS knockdown promotes the NF-κB pathway. (A-B) Western blot and qRT-PCR shows protein and transcription levels of SELS in primary hepatocytes and LMH cells. (C-F) Western blot and qRT-PCR were performed to detect the protein and transcript levels of NF-κB pathway in primary hepatocytes and LMH cells. All data were biologically replicated (*n* = 3). Bars labeled with different letters indicate significant differences screened by one-way ANOVA.

Considering that NF-κB is a rapid responder to noxious cell stimulation (Zambrano et al., 2020), we used the NF-κB inhibitor BAY11-7082 to assess the relationship between low SELS expression and the NF-κB pathway. The results show that inhibition of NF-κB after SELS knockdown further reduced SELS protein expression but did not affect its mRNA expression (Figs. 3A–B). Knockdown of SELS caused significant upregulation of the NF-κB pathway–related genes *NF-κB, TNF-α, TRAF6, PTGE, iNOS*, and *COX-2* in primary hepatocytes and LMH cells (p < 0.05), which could be inhibited by BAY11-7082 (Figs. 3C–F). These results indicate that SELS silencing activates the NF-κB signaling pathway and then induces the transcription of inflammatory signaling proteins.

### Inhibition of NF-κB attenuates SELS suppression–induced pyroptosis

**Fig. 4:**
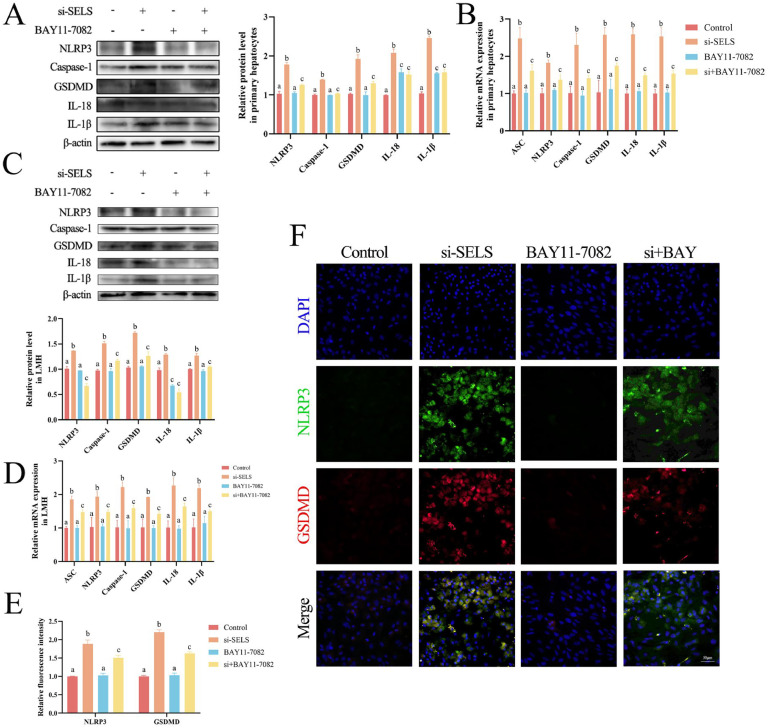
NF-κB is the upstream pathway of pyroptosis. (A-B) Western blot and qRT-PCR shows protein and transcription levels of pyroptosis-related genes in primary hepatocytes. (C-D) Western blot and qRT-PCR displays protein and transcription levels of pyroptosis-related genes in LMH cells. (E-F) Immunofluorescence images and quantification of NLRP3 and GSDMD in primary hepatocytes. All data were biologically replicated (*n* = 3). Bars labeled with different letters indicate significant differences screened by one-way ANOVA.

NF-κB normally promotes the transcription of IL-18 and IL-1β to mature pro-inflammatory cytokines released by pyroptosis (Yu et al., 2017). Therefore, we used the NF-κB inhibitor BAY11-7082 to assess the role of NF-κB in si-SELS–induced pyroptosis. We found that the addition of BAY11-7082 effectively alleviated the increase of pyroptosis-related gene protein and mRNA expression caused by SELS knockdown, including *ASC, NLRP3*, caspase-1, *GSDMD, IL-18*, and *IL-1β* in primary hepatocytes (Figs. 4A–B) and LMH cells (Figs. 4C–D). Immunofluorescence imaging showed that BAY11-7082 effectively reduced the fluorescence intensity of NLRP3 and GSDMD induced by SELS silencing, indicating that pyroptosis was alleviated in primary hepatocytes (Figs. 4E–F) and LMH cells (Fig. S2). These results indicate that NF-κB acts upstream of si-SELS–induced pyroptosis.

### SELS deficiency induces oxidative damage

**Fig. 5:**
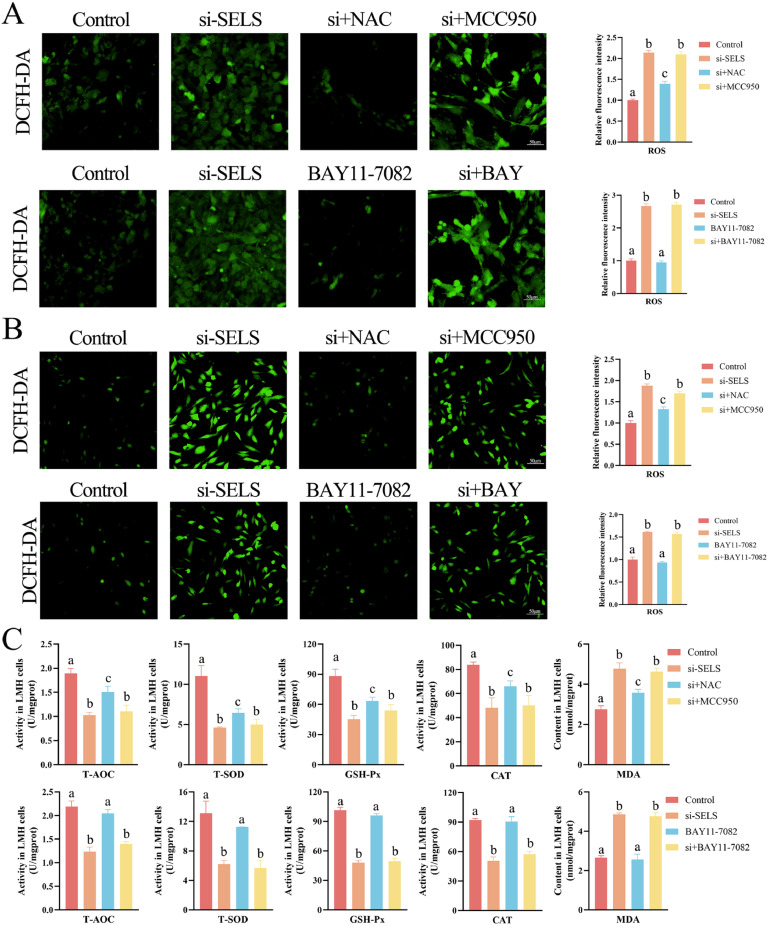
Evaluation of oxidative stress in hepatocytes. (A) DCFH-DA probe (10 μM) was used to evaluate ROS levels in primary hepatocytes. (B) DCFH-DA probe (10 μM) assesses ROS levels in LMH cells. (C) Determination of antioxidant capacity and oxidative stress levels. All data were biologically replicated (*n* = 3). Bars labeled with different letters indicate significant differences screened by one-way ANOVA.

The antioxidant effect of SELS on the liver was evaluated by assaying ROS production using fluorescence microscopy of the ROS probe DCFH-DA. Oxidative stress markers and antioxidant enzyme activities were detected after silencing SELS in chicken hepatocytes. The results indicated that SELS caused excessive ROS production and that NAC could effectively scavenge ROS (p < 0.05). Neither the NLRP3 inhibitor MCC950 nor the NF-κB inhibitor BAY11-7082 eliminated the ROS production by SELS silencing in primary hepatocytes (Fig. 5A) and LMH cells (Fig. 5B). Moreover, SELS knockdown significantly reduced the activities of T-AOC, T-SOD, GSH-px, and CAT in LMH cells, accompanied by an increase in the MDA content (p < 0.05). NAC addition restored the antioxidant capacity and reduced the oxidative stress levels; however, neither MCC950 nor BAY11-7082 alleviated the changes in antioxidant capacity and oxidative stress induced by SELS silencing (Fig. 5C). Thus, SELS inhibition in hepatocytes reduced the antioxidant capacity and triggered oxidative stress, independently of NF-kB and NLRP3 signaling.

### Pyroptosis results from ROS accumulation induced by SELS knockdown

**Fig. 6:**
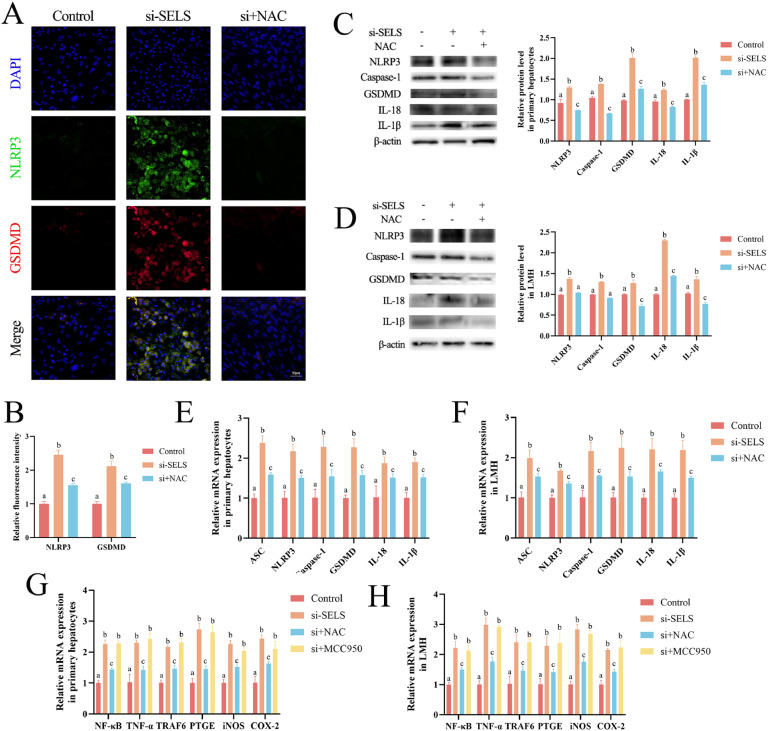
ROS regulates pyroptosis of hepatocytes. (A-B) Immunofluorescence images and quantification of NLRP3 and GSDMD in hepatocytes. (C-D) Western blot shows protein levels of pyroptosis-related genes in hepatocytes and LMH cells. (E-F) QRT-PCR detects transcription levels of pyroptosis-related genes in hepatocytes and LMH cells. (G-H) The mRNA expression of the NF-κB pathway in primary hepatocytes and LMH cells. All data were biologically replicated (*n* = 3). Bars labeled with different letters indicate significant differences screened by one-way ANOVA.

The ROS scavenger NAC was used to explore the relationship between oxidative damage and pyroptosis. We confirmed that ROS scavenging reduced the increase in NLRP3 and GSDMD fluorescence intensities caused by SELS knockdown. This was confirmed in primary hepatocytes (Figs. 6A–B) and LMH cells (Fig. S3). The increased expression of pyroptosis-related proteins caused by SELS knockdown in hepatocytes (Figs. 6C–D) was significantly alleviated by NAC addition (p < 0.05). In addition, the transcription levels of pyroptosis-related genes were significantly increased, and NAC reduced the pyroptosis caused by SELS knockdown (Figs. 6E–F). As described above, knockdown of SELS significantly increased the mRNA expression levels of *NF-κB, TNF-α, TRAF6, PTGE, iNOS*, and *COX-2* (p < 0.05) in primary hepatocytes and LMH cells, indicating that the NF-κB pathway was activated. NAC prevented si-SELS from activating the NF-κB pathway, whereas MCC950 did not (Figs. 6G–H). This implies that SELS suppression activates the NF-κB pathway via excessive ROS generation, independently of the NLRP3 pathway. Thus, SELS knockdown induced the release of ROS, thereby inducing pyroptosis.

## Discussion

Selenoproteins in the liver are essential for maintaining cellular function, and their expression is influenced by selenium availability as well as several physiological factors. Low selenium levels are associated with an increased risk of liver disease (Schomburg and Hughes, 2017). The normal metabolic activities of the liver are disrupted when selenium is deficient, triggering liver damage and fibrosis (Qiao et al., 2022). Selenium usually binds to the amino acid selenocysteine, further demonstrating its important role in the health benefits and biological functions of selenoproteins (Labunskyy et al., 2014). Thus, the roles of selenium and selenoproteins in liver diseases have received extensive attention. We found that selenium deficiency caused hepatocyte pyroptosis and decreased the transcript levels of several selenoproteins, of which SELS is a key regulatory protein. The oxidative damage induced by SELS knockdown in chicken hepatocytes was independent of the NF-κB and NLRP3 pathways. SELS knockdown activates the NF-κB pathway to induce pyroptosis, leading to the release of inflammatory factors and further exacerbation of liver injury.

Selenium and selenoproteins play regulatory roles in the development and progression of chronic inflammation. Selenium deficiency exacerbates the inflammatory response in calf intestine and mouse skin (Lei et al., 2023; Zhu et al., 2015). Selenium supplementation can reduce the levels of pro-inflammatory cytokines in rat liver (Al-dossari et al., 2020). Selenoproteins such as GPX1, GPX4, SELP, and SELS are involved in the release of inflammatory cytokines and free radical ions during inflammation (Hariharan and Dharmaraj, 2020). In this study, we confirmed that SELS ablation resulted in the release of inflammatory factors in chicken liver cells, indicating that SELS has a protective effect against liver inflammatory injury resulting from selenium deficiency. In addition, NF-κB acts as a transcription factor to regulate the activation and inhibition of inflammatory factors (Nettleford and Prabhu, 2018). NF-κB mediates the production of IL-1β and IL-6 to promote the recruitment of inflammatory cells and other inflammatory mediators (Feldmann and Maini, 2001). Inhibition of the inflammatory target genes of NF-κB is one of the effective treatment strategies for inflammatory bowel diseases (Papamichael et al., 2019). Other studies have shown that selenium deficiency increases NF-κB expression, whereas nanoselenium supplementation attenuates NF-κB protein expression in rat spleen (Xiang et al., 2024). In this study, SELS deficiency upregulated the expression of NF-κB related pathways and activated NLRP3 inflammasome assembly, promoting the release of the inflammatory cytokines IL-18 and IL-1β in chicken liver.

It is generally assumed that redox imbalance by ROS generation is related to liver disease. ROS promote the progression of chronic inflammation (Chen et al., 2024). After inflammatory skin injury in mice by selenium deficiency, the levels of the lipid peroxidation marker TBARS increased significantly and the activity of antioxidant enzymes was inhibited (Zhu et al., 2015). Daily supplementation with 1 mg/kg nanoselenium particles can attenuate sodium arsenite–induced renal inflammation and oxidative stress–related damage, and inhibit renal fibrosis in mice (Li et al., 2023). Li et al. (2018) showed that SELS silencing caused ROS accumulation in mouse hepatoma cells (Li et al., 2018). In this study, the accumulation of ROS and decrease in antioxidant capacity were more evident in SELS-knockout chicken hepatocytes. This finding suggests that SELS deficiency promotes oxidative damage in the liver. Selenium deficiency simultaneously produces ROS and activates NF-κB in dairy cow mammary gland (Zhang et al., 2022b). Cells exposed to H_2_O_2_ exhibited selenium-dependent alleviation of ROS accumulation and NF-κB activation (Kretz-Remy and Arrigo, 2001). In this experiment, the ROS scavenger NAC can effectively improve the redox imbalance and NF-κB mediated pro-inflammatory cytokine release in chicken hepatocytes. However, BAY11-7082 did not restore the oxidative capacity of hepatocytes. This indicates that the oxidative damage caused by SELS knockdown did not depend on the NF-κB pathway. This study found that SELS ablation promotes NF-κB activation to produce pro-inflammatory cytokines via a burst in ROS production.

Excess ROS can initiate pyroptosis in inflammatory diseases by disrupting cell membrane integrity and increasing the inflammatory response (Wang et al., 2024, 2021b). Selenium supplementation can attenuate NF-κB pathway-activated pyroptosis inflammatory injury associated with thyroid injury in rats (Zhao et al., 2024). Selenium deficiency initiates pyroptosis in chicken kidney cells, which is associated with the reduced expression of several selenoproteins and renal injury (Gu et al., 2022). However, yeast selenium can regulate selenoprotein levels to antagonize the upregulation of the NLRP3 inflammasome complex induced by heavy metal pollution (Xing et al., 2022). It has been reported that the development of liver fibrosis in mice can be reduced by blocking one of the three NLRP3 inflammasomes (caspase-1, ASC, or NLRP3). Similarly, blocking NLRP3 and IL-1β is one of the therapeutic strategies for the treatment of autoimmune hepatitis (Zhang et al., 2021). Miao et al. (2024) demonstrated that SELW regulates macrophages to promote pyroptosis of mouse hepatocytes (Miao et al., 2024). However, the relationship between SELS and hepatocyte pyroptosis remains unclear. This study showed that SELS silencing triggered NLRP3 inflammasome assembly, promoted the destruction of cell membranes by GSDMD, and the release of inflammatory mediators. A new function of SELS in liver inflammatory injury was confirmed. In addition, NAC, BAY11-7082, and the NLRP3 inhibitor, MCC950, effectively alleviated SELS-induced pyroptosis. However, MCC950 could not alleviate the oxidative damage and NF-κB pathway activation induced by SELS silencing. Taken together, we confirmed that SELS knockdown promotes NF-κB transcription by generating ROS, thereby inducing pyroptosis and inflammatory damage in chicken hepatocytes.

In conclusion, selenium-deficient diets decrease the expression of various selenoproteins, including DIO1, GPX1, GPX6, TXRD2, SELF, SELN, SELO, SELS, and SELT. SELS silencing causes excessive ROS production and oxidative stress, which in turn activates NF-κB pathway-mediated NLRP3 inflammasome assembly, thereby triggering hepatocyte pyroptosis and inflammatory damage. Taken together, these results reveal the mechanism by which SELS regulates liver inflammation and suggest potential targets for the prevention and control of hepatocyte pyroptosis and oxidative damage.

## Declaration of competing interest

The authors declare that they have no known competing financial interests or personal relationships that could have appeared to influence the work reported in this paper.

## Appendix Supplementary materials

Image, application 1

## Footnotes

Supplementary material associated with this article can be found, in the online version, at doi:10.1016/j.psj.2025.105269.
