# Supplementary material for: Selenoprotein S ablation-mediated pyroptosis contributes to liver damage resulting from selenium deficiency in chickens
Source: Poult Sci. 2025 May 5;104(8):105269. doi: 10.1016/j.psj.2025.105269 (PMC12142317; doi:10.1016/j.psj.2025.105269)
Supplement: Supplementary file 1 [file mmc1.docx]

**Supplementary material**

**Selenoprotein S ablation-mediated pyroptosis contributes to liver damage resulting from selenium deficiency in chickens**

Huanqi Zhang^a^, Xiaozhe Chen^a^, Tingjin Lu^a^, Qiyuan Cao^a^, Xiaojing Li^a,*^

^a^ College of Animal Science and Technology, Northeast Agricultural University, Harbin 150030, P. R. China

^*^ Corresponding author

All authors have read the manuscript and agreed to submit it in its current form for consideration for publication in the Journal

Tel: +86 0451 55191160

E-mail address: xiaojingli@neau.edu.cn (Xiaojing Li);

Table S1 Primers used for quantitative real-time PCR

| Gene | Forward Primer (5’-3’) | Reverse Primer (5’-3’) |
| --- | --- | --- |
| DIO1 | GCGCTATACCACAGGCAGTA | GGTCTTGCAAATGTCACCAC |
| DIO2 | ATTTGCTGATCACGCTTCAG | GCTCAGAAACAGCACCATGT |
| DIO3 | CTGTGCATTCGCAAGAAGAT | GCCGACTTGAAGAAGTCCAG |
| GPX1 | ACGGCGCATCTTCCAAAG | TGTTCCCCCAACCATTTCTC |
| GPX2 | ATCGCCAAGTCCTTCTACGA | ACGTTCTCGATGAGGACCAC |
| GPX3 | CCTGCAGTACCTCGAACTGA | CTTCAGTGCAGGGAGGATCT |
| GPX4 | CTTCGTCTGCATCATCACCAA | TCGACGAGCTGAGTGTAATTCC |
| GPX6 | TGCTCAGCAGACCCTAAAGC | AATAGGCGGCCACATTGACA |
| TXRD1 | TACGCCTCTGGGAAATTCGT | CTTGCAAGGCTTGTCCCAGTA |
| TXRD2 | GCTCTTAAAGATGCCCAGCACTAC | GAACAGCTTGAGCCATCACAGA |
| TXRD3 | CCTGGCAAAACGCTAGTTGTG | CGCACCATTACTGTGACATCTAGAC |
| SELF | ACTTGGCTTCTCCAGTAACTTGCT | GCCTACAGAATGGATCCAACTGA |
| SELH | CATCGAGCACTGCCGTAG | GACACCTCGAAGCTGTTCCT |
| SELI | TGCCAGCCTCTGAACTGGAT | TGCAAACCCAGACATCACCAT |
| SELK | GAAGAGGGCCTCCAGGAAAT | CAGCCATTGGTGGTGGACTAG |
| SELM | AAGAAGGACCACCCAGACCT | GCTGTCCTGTCTCCCTCATC |
| SELN | GCTGAATGAGCCACCAGACTTCC | CCACCTCCATGTTGCTGCCTTC |
| SELO | CCAGCGTTAACCGGAATGAT | ATGCGCCTCCTGGATTTCT |
| SELP | AGAACCTGAACCTGAACCAAGTGG | GATGGTGGTGGTGGTGTCTGTG |
| SELR | CGGTGCGGCTATGAGCTGTTC | TCGTGGATGGTCTCGGTGAAGG |
| SELS | CCGACATGGTGGTAAGAAGACA | GCTTGTGCATTCAACTCCTCTTG |
| SELT | AGGAGTACATGCGGGTCATCA | GACAGACAGGAAGGATGCTATGTG |
| SELV | GCTTCTTCCGCATTGGAGTCTGG | CTTCTCGGCAGCTTCAAGGACAG |
| SELW | CTCCGCGTCACCGTGCTC | CACCGTCACCTCGAACCATCCC |
| Sephs2 | TGGAACAAGATCAAGCTGGTGGTG | GAGCGTGGAAGGCGTGCATC |
| ASC | CTGGAGATGTGGTTTGGCCT | TTGGTTCTTGACCATCCGCA |
| NLRP3 | GCTCCTTGCGTGCTCTAAGACC | TTGTGCTTCCAGATGCCGTCAG |
| Caspase-1 | GTGCTGCCGTGGAGACAACATAG | AGGAGACAGTATCAGGCGTGGAAG |
| GSDMD | ACTGAGGTCCACAGCCAAGAGG | GCCACTCGGAATGCCAGGATG |
| IL-18 | TGATGAGCTGGAATGCGATGCC | TGGACGAACCACAAGCAACTGG |
| IL-1β | AGCAGCCTCAGCGAAGAGACC | CAGAGAAGGTCTGTCGAGTG |
| NF-κB | TCAACGCAGGACCTAAAGACAT | GCAGATAGCCAAGTTCAGGATG |
| TNF-α | CTCAGGACAGCCTATGCCAACAAG | GCCACCACACGACAGCCAAG |
| TRAF6 | ATAGCACGCAGCCTTGAGTT | GCATCAGCAGTGGCAGAAGT |
| PTGE | GTTCCTGTCATTCGCCTTCTAC | CGCATCCTCTGGGTTAGCA |
| iNOS | CCTGGAGGTCCTGGAAGAGT | CCTGGGTTTCAGAAGTGGC |
| COX-2 | TGTCCTTTCACTGCTTTCCAT | TTCCATTGCTGTGTTTGAGGT |
| β-actin | GCACCCAGCACGATGAAAAT | GACAATGGAGGGTCCGGATT |
|  |  |  |


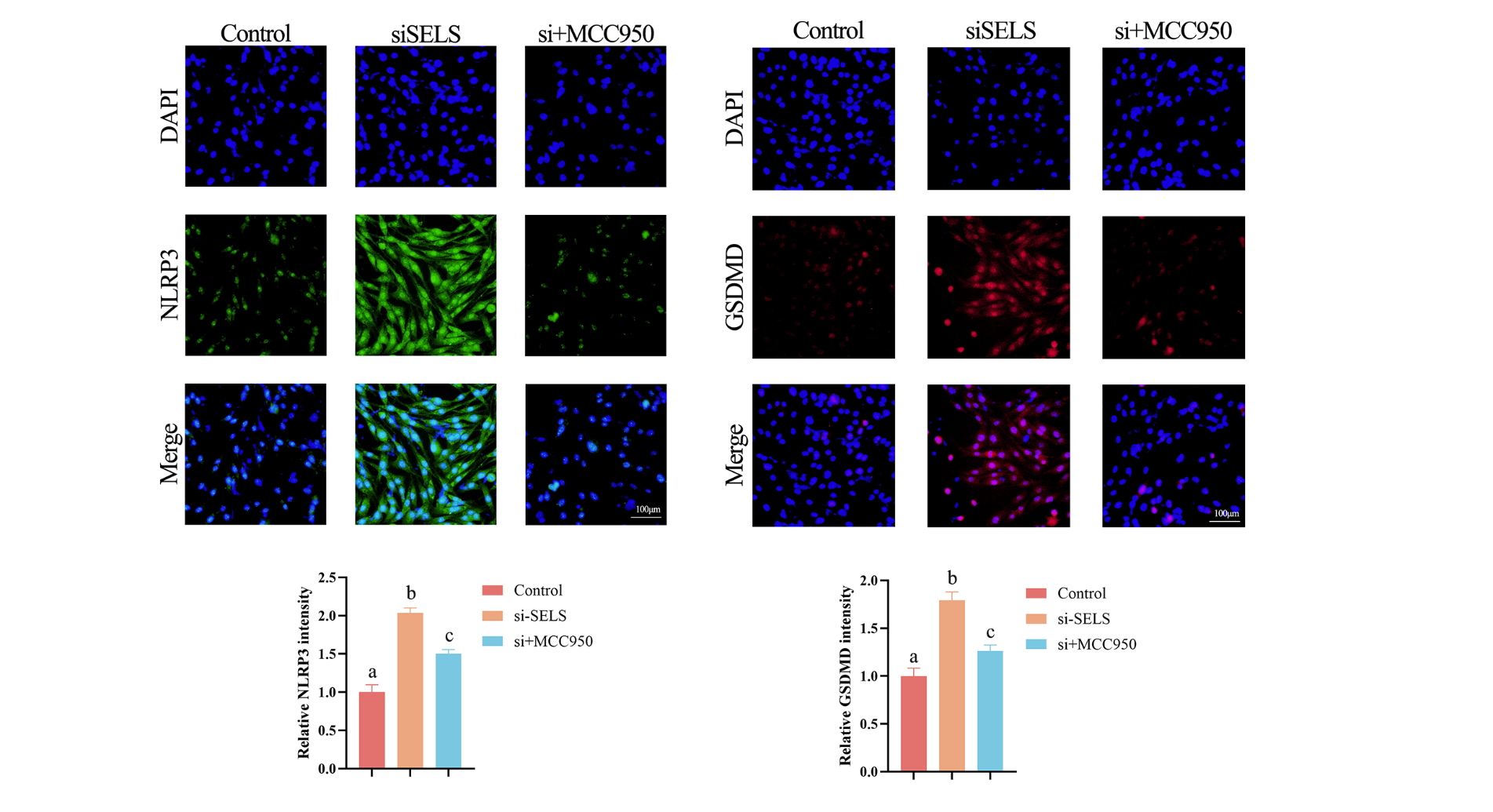


Fig. S1. MCC950 blocked si-SELS to activate pyroptosis in LMH cells. All data were biologically replicated (n=3).


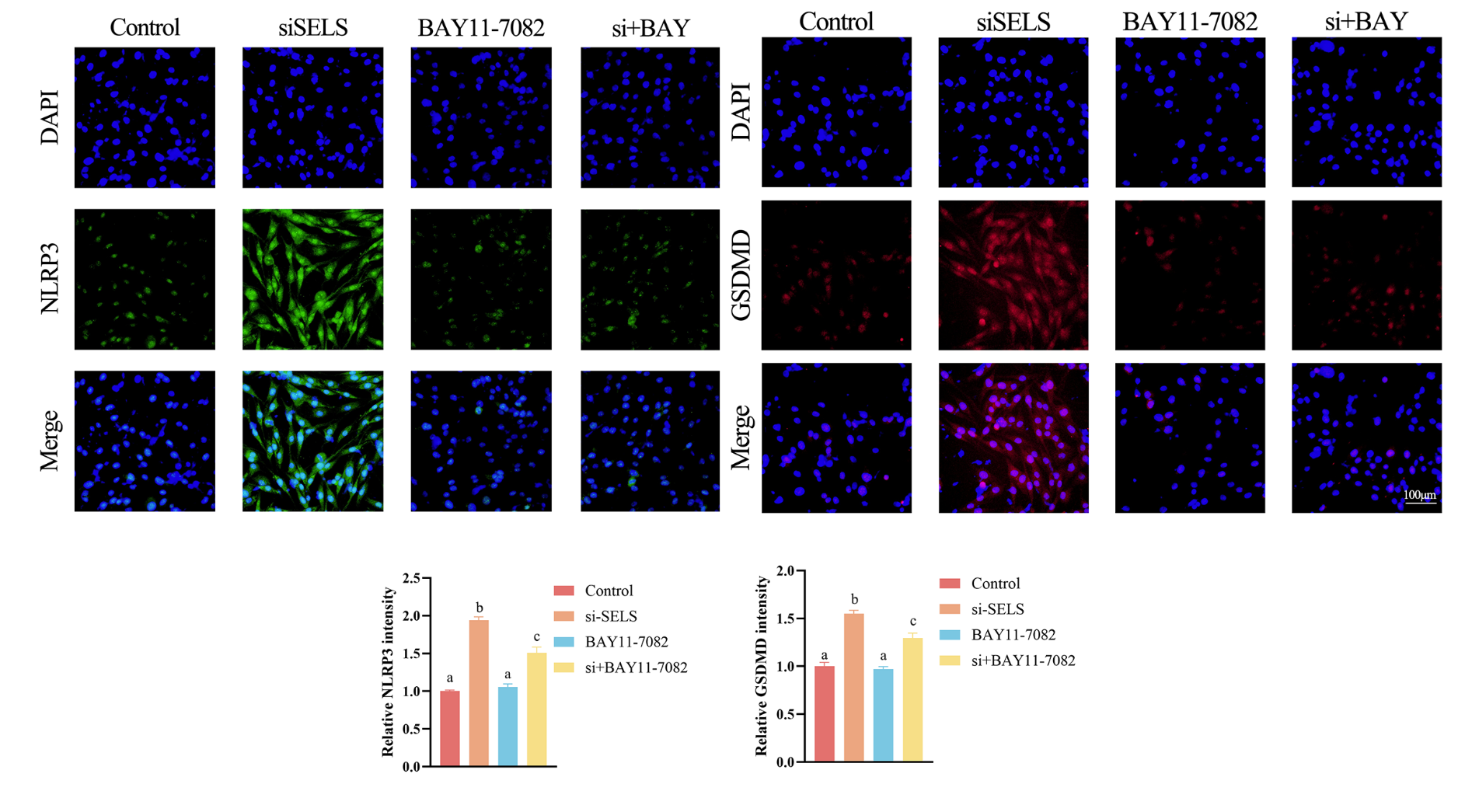


Fig. S2. BAY11-7082 attenuation of si-SELS-induced pyroptosis in LMH cells. All data were biologically replicated (n=3).


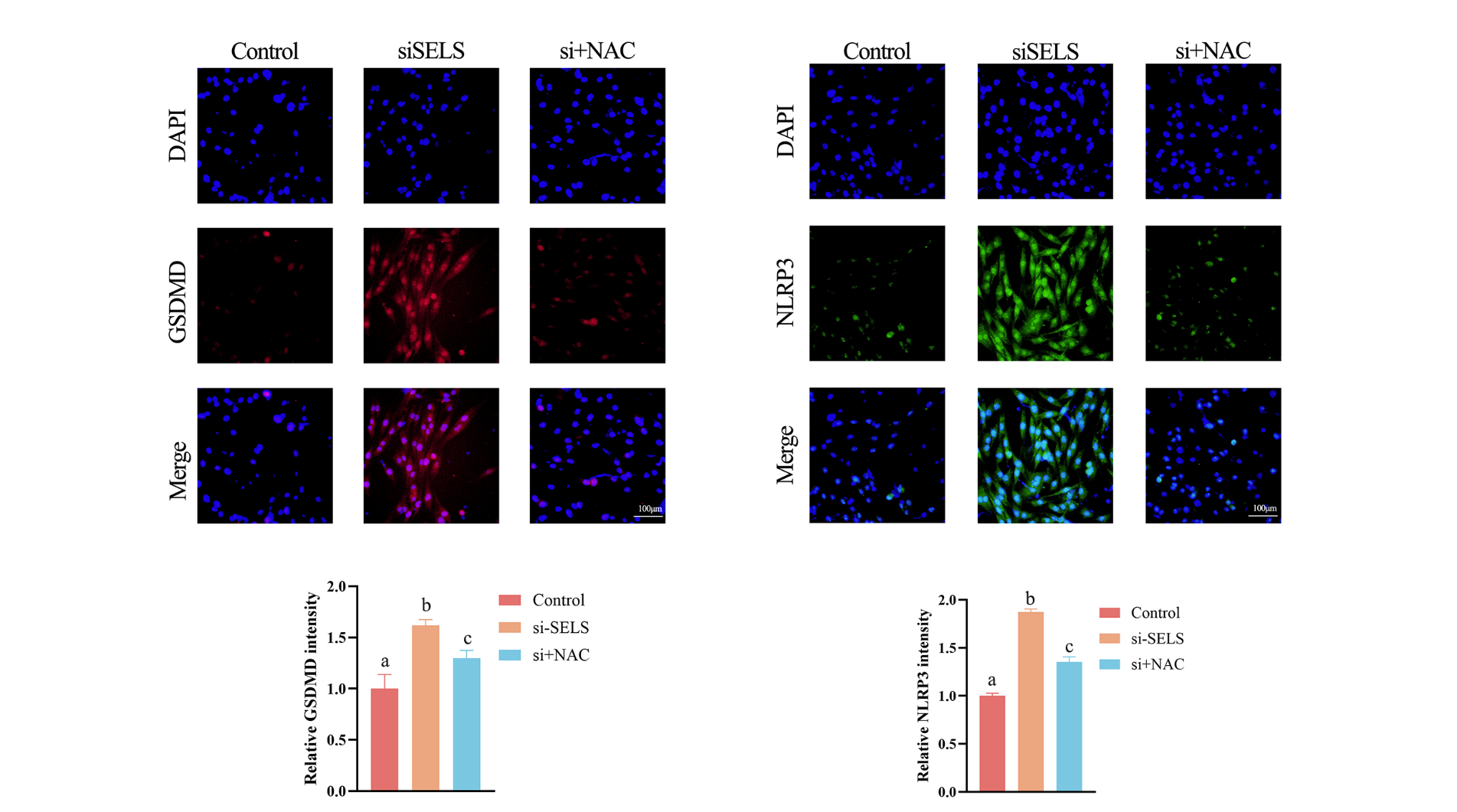


Fig. S3. NAC attenuated si-SELS-induced pyroptosis in LMH cells. All data were biologically replicated (n=3).
